# Remarks on the Pathological Condition of the System, Denominated Congestion

**Published:** 1828-11

**Authors:** John P. Harrison

**Affiliations:** Louisville, Ky.


WESTERN JOURNAL

OF THE

### MEDICAL & PHYSICAL SCIENCES.

NOVEMBER, 1828.

### ESSAYS AND CASES.

Art. I.—*Remarks on the Pathological condition of the System, denominated Congestion.* By John P. Harrison, M. D. of Louisville, Ky.

Independent of the inherent difficulty which attaches itself to every recondite subject of inquiry, there is often a very great hinderance presented to the investigation of some topics of science, by the imprecision and ambiguity of language. Words, being but arbitrary signs of our ideas, are liable to a variety of significations, according to their derivative import, or conventional meaning. As an illustration of this remark, the word Congestion may be adduced. According to its derivation, it means, to heap up or gather in a heap, from the Latin word *congero;* and Boerhave, says Dr. Parr, employed it to denote the accumulation of blood in a part, produced by lentor, or spissitude of that fluid. By Dr. Cullen it is used to express the fact of increased action, or inflammation of a part. Dr. Armstrong, the most enlightened advocate of the doctrine of Congestion, and who may be considered as the master spirit that has moulded it into some form, and given to it the strong attractions of a fascinating theory, employs the term, evidently, in a signification rather mechanical. It requires no very comprehensive retrospection over the path of medical history, to enable us to see the injurious effects which have resulted from the introduction of terms borrowed from mechanics, hydraulics, or chemistry, to explain the actions of the living animal economy, whether in a healthy or morbid condition. Figurative language, when employed to embellish moral subjects, is understood, agreeably to the nature of the subject of such embellishment. But when a term, used in one department of natural science, is transferred to another, confusion and misconception are apt to be engendered; and the idea *originally* communicated by such a term, is constantly associated in the mind with its transferred employment. Thus, not to multiply a great variety of instances, the word Congestion, which is a mechanical phrase, expressing a quality of an inert mass, is used by many medical writers, to denote a mechanical accumulation of blood in a part of the living system. The physiological laws of the economy are lost sight of, and merged in the unvarying and immutable condition of a phenomenon, which, properly regarded, is but an effect.

Accuracy and precision in scientific terminology subserve three important ends. First, a copious and definite techniology is an instrument of intellectual analysis. Secondly, we discriminate with greater perspicuity the objects of research, when we have a distinctness of view kept up in the mind by a determinate language, designating such objects. Thirdly, to impart our thoughts to others, the utility of a nomenclature, replete with a diversity of meaning, and yet accurate in the import of each word, is universally recognized. But when a word, such as Congestion, is employed in both a generic and specific sense, without proper regard to the pathological state of the system, or part, under deranged action, error must inevitably spring up from this source, and the judgment of the practitioner be misled in its decisions on the momentous concern of a fellow being's life. That I may not transgress against the spirit of truth, by which our minds alone should be guided, I will copy from Dr. Armstrong his own views on the subject, to show the reader the discrepancy and confusion which existed in that eminent physician's mind, on the pathological state of the whole, or a part of the system, called by him Congestion. I will in the next place endeavour to point out the danger of mistaking four pathological states of the whole, or part, of the system for Congestion—viz:

1st. Sympathetic derangement.

2d. Constitutional irritation, or shock.

3d. Suffocated excitement.

4th. Collapse of the vital powers in the last stages of fever. And the discussion will be closed by some general and practical reflections.

The following quotations from Dr. Armstrong, go to prove that some degree of confusion existed in his mind on the pathological doctrine of Congestion.

"In congestive cases, the local accumulations of blood in the veins obstruct from the beginning, the common series of febrile phenomena, and there is in consequence either a total want of morbid heat, or a concentration of it in some particular parts of the body, whilst others are considerably beneath the natural temperature. It is the entire absence, or partial presence of excitement, which constitutes the chief external distinction between the severest forms of the congestive typhus; as they all coincide in oppressing the functions, or in deranging the structure of some important organ, by an *almost stagnant accumulation of blood in some part of the venous system.*"*

Attacks of the worst cases of congestive typhus, says Dr. A., are sudden, and marked by "an overpowering lassitude; feebleness of the lower limbs; deep pain, giddiness, or sense of weight in the encephalon; a dingy pallidness of the face; anxious breathing; damp relaxed, or dry withered skin; and those peculiar conditions of the temperature which have been noticed above. The pulse is low, struggling, and variable; the stomach irritable; frequently there is an inability from the first, to hold up the head; and the mind is more affected with dulness, apprehension, or confusion, than with delirium. The whole appearance of the sick impresses the attentive practi-

* Practical Illustrations of Typhus Fever, p. 73.

tioner with the idea, *that the system in general, and the brain in particular, are oppressed by some extraordiuary load.*"

The reader would infer from reading the above extracts, that the eminent author had very definite and accurate conceptions with regard to the symptomatology and pathology of Congestion. That in the author's own words, it was clear, "arterial excitement was an excess, venous congestion a deficiency, of natural action," and that there was a morbid arrestation of blood in the veins, giving rise to a preternatural accumulation, amounting almost to an entire stagnation of the vital current. And yet, Dr. A. in his obervations on Pulmonary Consumption, says, "It necessarily follows that when the energy of the heart and arteries is much diminished, that they cannot maintain the natural current of arterial blood, and of course a proportionate accumulation takes place in the veins; *and this venous accumulation appears to load and stimulate the capillaries of the arterial system, by retarding the return of the blood through them.*"—p. 159.

Now in accordance with Dr. A's own views of increased action, or inflammation, Congestion is inflammation itself, as we read at page 288, of his work on Typus, under the head of Common continued Fever. "The action of one artery, says he, I have never known greater than that of another, and *what we call increased action is, I suspect, merely increased accumulation, and what we call increased determination is, I also suspect, merely an increased volume of the vessels arising from an impediment to the return of the blood from the quarter to which those vessels lead.*"

From the above quotations, it is very obvious, that he had no distinctly marked line of difference in his own mind, between inflammation and Congestion, but that agreeably to his own words, the pathological condition of the parts was identical. Dr. Potter, his distinguished American annotator, concedes all that we could desire on this point. In a note at page 67, he says, "The observant author has accurately depicted the phenomena of the congestive state, but there is great difficulty in explaining, on the known principles of pathology, how venous congestion can occasion such deplorable consequences, without the instrumentality of arterial action. Although the first impression of the action of the heart may be transient, it is often violent, often distends the distant smaller ramifications of the arteries, and sometimes ruptures them. In a subject that was examined by the knife at my request, one that had been well characterized by all the prominent symptoms of venous congestion, as they are described in the text, the arteries of one of the kidneys, a part of the peritoneum, and a considerable portion of the intestines were evidently inflamed, while the extraordinary distention of the veins was remarkably perspicuous."

The illustrious Gregory has said with truth, *Medicina enim neque agit in cadaver, neque repugnante natura aliquid proficit,* that medicine will neither act on a dead body, destitute of vital property, nor will it act *contrary* to the *laws* of organized animal existence. Now what is true, as it respects therapeutics, is equaliy true in reference to pathology. There can be no pathological state of a part, or the whole body, generated by a process exactly similar to the method in which mechanical, or chemical results are obtained. This is the rock on which Dr. Armstrong's frail theory is wrecked. Here is the fallacy on which he built his hypothesis. If we for a moment contemplate the moving powers of the circulation, and keep incessantly before the mind the laws which govern the animal economy, the irresistable conclusion will fasten upon our judgment, that the theory of venous congestion independent of arterial impulsion, is altogether vague and ambiguous. The heart is the great central impellent organ of the blood in the entire march of the circulation. An uninterrupted continuous column of blood fills the pulmonary, as well as the systematic, arterial, and venous tubes. The pulse which we feel, when our fingers are laid on the radial artery, is not occasioned by any dilatation of the calibre of the artery, but the stroke, or impulse which the column of blood receives from the heart, resists the pressure thus made, and in this pulsation the arterial tube is passive. We consider it very doubtful whether the capillary system has any power of independent circulation. But this physiological question, replete as it is with curious and interesting matter, I must pass over. The venous system has no endowment of function which would enable it to act without a previous exertion of power by the heart.

Although the automatic diastole of the auricles, assisted by the pressure of the circumambient atmosphere, muscular contraction, and the elasticity of the veins, may all concur in assisting the circulation of the blood in the veins, yet the power of the heart is the main cause of the progression of the vital fluid through the entire tract of both the arterial and venous systems. Admitting these facts, which the late physiologist in England and on the continent of Europe have irrefragably established, we see at once the fallacy connected with the theory advanced by Dr. Armstrong, and so generally adopted by physicians. Suppose the action of the heart is weakened, what results? Not an accumulation of blood in the venous tubes, as Dr. A. asserts, but the column of blood which pervades alike, both the arteries and veins, is not so forcibly impelled towards the extremities. The diminution of action must alike pervade the arteries and veins.

The power of the heart is altogether a vital power: but the process by which it accomplishes the final results, the ultimate end, that is, the circulation of the blood, is brought about by the agency of a mechanical arrangement in the vessels. Now, let us take two sets of tubes, imitating the arteries and veins, made of some elastic matter, and at the centre of the entire circle, place a piston. It is manifest that when the piston is forcibly impelled against the column of fluid which fills them, an impulse must be conveyed throughout the whole circle. That there are certain vital properties with which the capillary system of vessels is endowed, is proved from the known fact, that all the secretions are performed by that system. Instead of saying with John Hunter, that the blood contains the principle of animation, we might safely lodge it in the capillary vessels. But these have enough to employ them when engaged in the important vital processes of secretion, in all its diversified results, without taking any part in aiding the heart, to propel the blood onwards through them.

The appearances seen in an examination of the dead body, are adduced by Dr. A. as corroborative of his views of venous congestion. But it is a well known fact, that in a majority of cases, the blood is invariably found in the venous system, the elasticity of the arteries forcing it into the veins, and the lungs ceasing to expand, the blood accumulates in the pulmonary artery, and consequently, a reflux of its current arrests that fluid in the right auricle. When an animal dies suddenly, the functions of the brain and heart being destroyed, without the intervention of the lungs, the venous system is not found so loaded with blood.

The brain, liver, and intestines, are the organs most liable to the state, called venous congestion. The structural economy of the brain, as respects the circulation of blood through it, is well adapted to prevent the evils of venous congestion. In cases of violent muscular effort, when respiration is suspended, as in parturition, and every vessel about the head seems full, almost to disruption, no injury results. The sinuses of the brain may be distended with blood, and yet, from their position, no pressure is induced on the cerebral mass. The pia mater, tunica arachnoides, or substance of the brain itself may be the seat of acute or chronic inflammation. M. Martinet, in his Pathology, says, "permanent congestions of the pia mater, encephalitis, and ataxic or nervous fever, may be mistaken for arachnitis of the convexity of the brain; dropsy of the ventricles, 'ramollissement,' or softening of the hemispheres, of the corpus callosum or cerrebellum, and adynamic or putrid fever, may be mistaken for that of the base, and finally, hydrocephalus, and several chronic alterations of the brain, may be mistaken for chronic arachnitis."—p. 127.

The symptoms indicating what part of the encephalon, or its investments, are inflamed, seem very equivocal, and are not to be confided in, as of much moment. The treatment in all such cases is similar. From its being the general receptacle of all sensitive impressions, as well as the point of conveyance for the various irritations of automatic life, the brain is often functionally deranged, as will be shown. The liver is considered by some medical gentlemen as a sort of *officina morborum*, the fountain head of all diseases. Dr. J. Johnson, in his Tropical Climates, has strenuously insisted, that congestion of the liver is the proximate cause of dysentary and bilious fever, the remote cause being the injurious agency of a mutable temperature on the skin, propagating a morbid action to the portal circle, through the cutaneo-hepatic sympathy. But yielding to Dr. J. the specious physiological law of a cutaneo-hepatic sympathy, as exercising a dominant control over the liver, yet how can we account for the subsequent symptoms of either dysentery, or bilious fever, upon such grounds of explanation.

From the structural arrangements of the liver, we perceive it subserving a two fold office. It is a secretory organ, exerting a very extensive influence over all the functions of the other abdominal viscera. And as a transmitting organ, through which the black abdominal blood finds its way to the heart, its importance in the economy is enhanced. Broussais, in his Physiology, has demonstrated that the portal circle acts as a diverticulum, or reservoir, for the blood when the action of the heart is much hurried, or a retrocession of blood takes place from the general surface of the body. The venous blood is arrested, before it reaches the heart, in the vena portæ, and thus the heart is not suddenly overwhelmed by too great a supply. This eminent physiologist, contends "that the vena portæ is always engorged in those who die of gastro-enteritis. Though it is evident, the effect has been received as the cause, since it is irritation of the intestinal mucous surface that accumulates blood in the veins of the abdomen."—p. 388.

I cannot impart more information to the reader, who has any doubts on this point, than by copying from Mr. Shaw's Manuel of Anatomy, the result of his very extensive observations. "It is a very common mistake to describe the loaded state of the vessels as an appearance denoting inflammation; the state of the true inflamed intestine is so distinct, that it can hardly be forgotten after it has been once seen. In the first stage there are numerous small vessels seen, upon the gut, like those on the eye in opthalmia, with a suffusion around them; in the second stage there is matter, or lymph, effused; and in the more advanced state, adhesions are formed between the surfaces of all the intestines."—p. 65.

"In the greater number of those who die of fever, the intestines appear gorged with blood not inflamed; but on opening the lower part of the small intestines, we shall generally discover small ulcers, with thickened edges; this appearance is almost always found in the great intestines of those who have died of dysentery."—p. 67.

The limited space allotted by me, forbids an indulgence of annotation on the above remarks of Broussais and Shaw—let the reader reflect on them, and deduce his own conclusions.

My next object will be to point out those affections which wear the guise of venous congestion,—agreeably to Dr. Armstrong's doctrine on that subject. The first class of cases, which simulate the symptoms of venous congestion, according to the order of the arrangement adopted, comprises all of those affections which are produced by sympathetic irritation. As a few apposite facts are of more value in settling controversial points, than a multitude of reasons, however ingenious, I will notice several veritable instances of the agency of morbid sympathy, in occasioning symptoms of a threatening character to life.

I was called, in the autumn of 1822, to see a boy in convulsions. He was of a spare habit, about eight years old, and of rather a feeble constitution. He was laboring, as I then thought, under congestion of the brain. His skin was rather cold, pulse quick and tense, and bowels costive. I took from his arm about a pint of blood, but there was little abatement of the convulsions. A blister was applied behind the neck, and ten grains of calomel given as soon as he was able to swallow.

The case seemed hopeless, and I left the house under a firm conviction that venous congestion in the brain was doing its work of death, in defiance of my applications. The fits did not abate till the calomel operated, which it did in three or four hours, being assisted by stimulating enemata. When it operated, a large handful of ascarides were evacuated, upon which the convulsions left him entirely.

Whilst a student in the year 1815, I was sent to see a child with all the symptoms of hydrocephalus—strabismus was present. This child was cured by one dose of calomel, which brought away a considerable number of lumbricoides. Cases analagous might be multiplied, but as the above are not as directly applicable to our subject as the reader may demand, I will give two others from my own observation, and one from Dr. Marshall Hall's work on Mimoses.

In the summer of 1819, I was called to see Mrs. F. in a state of coma, with reduced arterial action, coldness of surface, and constipated bowels. As I had never seen her before whilst thus attacked, though I understood she was subject to similar seizures, my mind was much embarrassed concerning the nature of the malady. But after much inquiry of her friends, and a careful inspection of the case, I concluded it to be a nervous affection, resulting from a sympathetic irritation of her stomach and bowels. I ordered injections of a strong solution of common salt and water, which operated and produced some reaction of the heart. As soon as practicable, she took a gentle emetic, which excited a pretty copious vomiting of mucus—after the action of the emetic, her muscular system became much irritated, and decided symptoms of hysteria were manifested. Sulphuric æther and laudanum were given to calm the excessive irritability of the system, and the convulsions, and other hysterical symptoms soon left her.

Here the irritation was lodged in the stomach and bowels; perhaps both had suddenly fallen into a state of atony and inaction, which gave rise to symptoms like those of venous congestion.

The following case is one of the most interesting of the kind I ever saw. Mr. H. sent for me in July, 1827, and the young gentleman who came, informed me that the attending physician was out of town, and that such was the unexpected change for the worse in Mr. H's case, that he required my immediate attendance. I immediately waited on him, and found him with cold extremities, clammy skin, feeble pulse, and great restlessness. Very soon, a sensorial disturbance supervened, and he became slightly comatose, which in the course of the night augmented into a complete suspension of all consciousness.

All these phenomena were induced by drinking ice water, whilst convalescing from an attack of dysentery. At first he vomited some dark colored, flocculent, mucous fluid, but after giving him a dose of laudanum and aq. amm. pur. the irritability of stomach subsided.

Dr. Ferguson, his physician, a gentleman of known skill in his profession, and myself remained with him till twelve that night. When we left him, he was still sunk in a profound state of insensibility, his pulse scarce perceptible, his skin cold and bathed in a profuse clammy sweat. Sinapisms were applied to his ankles, and the laudanum and aq. amm. pur. with wine whey, were continued through the night. We left him under a full persuasion that he was moribund. But next morning, the appearances were more auspicious, yet there was no return of consciousness. The course of stimulation was persevered in through this day, and the next night, and on the third day, there was a manifest amelioration of all the symptoms—in a week he started home in a steam boat, but died on the voyage, of dysentery, after being detained on board five days longer than he expected, from an accident happening the engine.

Here we see a series of morbid movements excited in the action of the heart, in the functional powers of the brain, and in the skin, from the excessive irritation of cold water on a weak and irritable stomach.

It is a familiar fact, that when a man drinks a quantity of cold water after exhaustion from muscular exertion in a hot day, his mind soon becomes deranged, and he sinks rapidly under the prostrating agency of the injury, unless ready aid is afforded. Here the excessive irritation acts on the nervous system through that organ, which Mr. Hunter has said is "the seat of simple animal life, and thereby the organ of universal sympathy of the materia vitæ, or the living principle."*

* Hunter on the Blood.—p. 361.

Dr. Marshall Hall, in his valuable little work on the Mimoses, says, the Mimoses Decolor, with affection of the head, is, in many instances, mistaken for sudden tendencies or congestions of blood with regard to the brain, and for insiduous or chronic inflammation of this organ.

"*Case* LXXI.—Mrs. C. of B. aged 45. She is affected with the mimoses decolor, &c. This lady has for many years been subject to attacks of *violent pain of the head*, accompanied with a sense of constriction about the neck, for which *bleeding*, *blisters*, and a *seton*, have been variously recommended by various practitioners. The affection continued to recur, notwithstanding, for very many years, always inducing the fear of some attack of an apoplectic nature. It has yielded however, to a *persevering* use of efficient purgative medicines." In another case, the patient was treated first for an affection of the brain, and then of the liver, "by bleeding, leeches, and blisters, to an almost incredible extent."—p. 153. The skin in the above cases was of a dingy aspect, and there was much prostration.

I have seen a man operated on for fistula in perinæo, in whom rapid appearances of venous congestion arose, when the urethra was cut. Irritation of the urethra from the introduction of the bougie, at times, brings on an attack of shivering, with other symptoms belonging to Dr. Armstrong's congestion.

I trust that I have made apparent to the reader's mind, the important clinical fact, that a simple irritation of a viscus, or other sensitive portion of the body, may by a sympathetic extension of such irritation through the general system, induce those phenomena, which are laid down as characteristic of congestion of blood in the veins. It is not contended that all the symptoms enumerated by Dr. A. are present in such cases. Neither are they always presented in high relief in those instances, in which Dr. A. would contend for the existence of that pathological state, which I am now controverting.

The second class of simulated venous congestion cases, is composed of the varieties of *constitutional irritation*, or *shock*.

Such is the copiousness of this part of my subject that great compression of thought is demanded, to keep within the bounds of a desirable brevity. I cannot here, perhaps, act more judiciously than in giving a valuable extract from Mr. Travers's master-piece of medical surgery, his "Inquiry concerning that disturbed state of the vital functions usually denominated Constitutional Irritation."

"A patient in a case of fatal burn, after the first expressions of anguish have subsided, nearly resembles a person stunned by a fall, or as much stupified with liquor, without suffering an actual suspension of his senses. Inspection demonstrates fulness of the veins of the brain, and its membranes, and effusion beneath the arachnoid membrane, confirming the symptoms which occur in the latter stage. But these appearances are too slight, and too frequently seen in the bodies of persons who die rapidly, to be much dwelt upon. The proximate cause of death appears to me to be a species of concussion, functional, not organic, by which the brain is deprived of its influence over the organ of circulation; for the symptoms of cerebral disorder are first manifested; secondly, a diminution of the power of the heart; thirdly, the respiratory function becomes impeded, as a necessary consequence of the two first."—p. 80.

The following case came under my observation in the fall of 1821,—it is a good exemplification of constitutional irritation from loss of blood. The symptoms so nearly resembled those laid down by Dr. Armstrong, as characteristic of venous congestion, that had I at the time not known the *cause* which produced them, my mind would not have paused a moment in arriving at the conclusion that great accumulations of blood in the veins of the vital organs, especially the brain, almost to a "stagnant plenitude," as Dr. A. would say, existed, and were the real *fons et origo* of all the morbid phenomena.

A poor woman, in the seventh month of her pregnancy was attacked with uterine hæmorrhage. Supposing it to be the result of too much labour, I ordered her to be quiet in bed, and gave her an opiate. It returned in twelve hours after the first eruption, with fearful violence.

My friend, Dr. J. C. Johnston, a gentleman of accomplished attainments in his profession, was sent for to assist in the delivery, which I now saw to be essential to the preservation of her life. We proceeded to the task. The placenta was found attached over, or around the mouth of the uterus—the blood was pouring forth in one unabated current; the pulse began to flag; the woman became delirious; her extremities were getting cold, and the general surface assuming a shrunken aspect that betokened the rapid exhaustion of the system. In this state her mind wandered to a great degree, and it was with some difficulty that we could induce her to submit to the delivery of the child by turning.

After a persevering and toiling effort of several hours, we accomplished the work by gradually dilating the os tincæ, working the hand up between the placenta and uterus, breaking the membranes, and bringing down the feet. The explicit directions given by that eminent teacher of Midwifery, Dr. Dewees, we found of invaluable benefit in guiding us to a correct treatment of the case.

The patient was now entirely pulseless, and there was little prospect of saving her life. But we gave her a dose of laudanum, with brandy toddy, to calm the constitutional irritation, and left directions to continue the stimulant plan through the night.

Reaction took place in the morning, it being midnight when we left her. Still the head was affected; there was some confusion of mind, and much throbbing in the arteries of the head. Quietude was enjoined, and cold applications to the head were used. Towards evening a purge was given, and the cold applications continued. In a week she was convalescing, and had a slow recovery.

The following case is from Mr. Travers's work. "A man fell from the roof of a coach, and in the fall suffered a compound fracture of the leg. He was replaced on the coach, and brought to London, a distance of forty miles. On his arrival at the Hospital, he appeared as if intoxicated, but recovered from this state in a few hours. On the third day, without any considerable previous inflammation, the leg began to assume a gangrenous appearance. On the fourth day he became insensible: his breathing stertorous, and the pupils of his eyes dilated. The symptoms so much resembled apoplexy, that it was supposed some effusion of blood had actually taken place in the brain. The same night he died, *and on examination, no morbid appearances whatever was found in the brain or other viscera.*"—p. 82.

In a paper which I published in the 1st No. of the Transylvania Journal of Medicine, on Cholera Infantum, I have attempted to show, that when children die with symptoms of hydrocephalus internus, after a violent seizure of cholera, the symptoms, imitative of the cerebral affection are only functional disturbances of the brain, produced by the shock imparted to the nervous system, from the violent intestinal malady.

Mr. Hunter says he has seen tetanus from loss of blood. Any agent which makes a sudden breach upon the functional integrity of the nervous system, will occasion the symptoms of constitutional irritation. Sudden and great losses of blood in irritable constitutions, especially in children and women; injury done a limb; surgical operations; protracted cold to the surface, where the powers of resistance are weak; febrile excitement; mental emotions, &c.

I have repeatedly seen men die of fever, when there were evident manifestations of cerebral affection, such as dilated pupils, delirium, subsultus tendinum, optical illusions, and even convulsions, and yet upon examination of the brain after death, no organic læsion whatever was seen.

The third pathological state with which venous congestion may be confounded, is *suffocated excitement.*

Dr. Rush introduced this term, and intended to import by it the fact of a high degree of inflammatory action, creating a partial suspension of the functions of life, from its intense and overmastering influence of the system.

Sydenham pointed out the important practical truth, that the petechiæ and vibices sometimes seen in fever, do not proceed from debility giving rise to a mechanical exudation of blood under the skin, but are the evidences of "nature's being in a manner oppressed and overcome, so as not to be able to raise regular symptoms, adequate to the violence of the fever; all the appearances being quite irregular."

Dr. Rush, whose glory lives in the imperishable monuments of genius, which his own hands erected on the field of American Medicine, acknowledges his obligations to the English Hippocrates for his views on this point. It was the defect of our illustrious countryman's pathology, that it was too exclusively confined to the blood vessels.

Dr. Parry, of Bath, in his able work on Pathology, &c. has followed Dr. Rush, with some enlargement, and a more precise enunciation of seminal principles, but his pathological doctrines are amenable to the same objection. It appears that Dr. Makittrick, in 1772, published his Commentaries on the Principles and Practice of Physic in Bath, and promulgated the same leading views which the elder Parry, in 1815, gave to the world.* It is the more necessary to warn the reader against an unqualified reception of the pathological systems of Rush and Parry, because they are so popular, from their simplicity and comprehensiveness.

Every practitioner of medicine should be like the judicious Boerhave—I mean judicious in this respect—eclectic; carefully sifting and analyzing every opinion, and giving a due appreciation to every well recorded fact. The man that bows his understanding to any popular opinion, regardless of its harmonious adjustment to the order of natural phenomena, may become skilled in *adopted* reasoning, but will never have a judgment well balanced, nor a memory well stored with facts.

With these introductory remarks to the third head of my subject, I will now give two cases, reserving practical inferences, agreeably to my plan, for the concluding remarks of the general discussion.

A poor woman sent for me in 1822, during the prevalence of a desolating epidemic bilious fever; not being able to go, I sent her a dose of calomel and jalap. Next day, understanding that she was worse, I visited her. Upon my

* Western Medical and Physical Journal, Nos. I. and IV.

arrival at the house, I understood that she had been delirious all night, and had attempted to get out of the window. This was the third day of her fever—her skin was cold and clammy, her pulse soft and compressible, with very little force in the stroke of the heart; her tongue was brown; and on her arms and breast were petechiæl spots. She had vomited some dark coloured fluid, and her bowels were constipated. Death was evidently approaching rapidly. I attempted to draw some blood, but the vein soon collapsed, after a very little was detracted. Warm drinks, with sinapisms, were ordered, but she died in a few hours.

No inspection was made by the knife, but it is evident that the stomach was the suffering organ. Could mere venous congestion of that viscus so soon destroy life? The answer may be yes, if by venons congestion you mean that phenomenon which the veins present in inflammation of a part—that is, increased fulness, and augmented volume.

In the winter of 1828, I was invited to see the post-mortem examination of a negro man, who had died about eight or ten hours before we arrived at the house. His physician gave the following summary of his attack, and the treatment pursued.

On Friday he was employed in getting some lumber out of the Ohio, which had inundated its banks, and threatened to carry off every thing near its shores. He drank a good deal of whiskey, was very wet all day, as he had to wade in the water for the plank, &c., and worked hard till late in the evening. That night he was taken with a chill, when the physician was sent for. He immediately gave him calomel. Next morning the skin was still cold, and pulse feeble. He was purged. This day, or the next, a small quantily of blood was drawn. The treatment pursued afterwards was calomel, opium, and camphor, in large quantities. The patient evacuated blood for two days before death. In one week the case terminated in death, previous to which event there was stupor for two days.

There were five physicians at the examination. Three of them were veterans in the warfare with disease, two were gentlemen who had practiced nine or ten years. Two of the older gentlemen pronounced the case to be a genuine example of venous congestion. The others thought that inflammation would be found in the bowels. The brain was examined, there was no appearance of inflammation about it, or its meninges, but the two *congestion* physicians thought they discovered venous congestion on its surface, as there was evidently dark coloured, (not quite black) blood found in the sinuses of the encephalon. The three *inflammation* gentlemen saw no congestion about the organ.

The thorax being opened, all the viscera in that cavity seemed in good keeping; but there was some darkness of aspect about the portion of the lungs that lay towards the spine. Here was fresh matter for a contrariety of opinion. Congestion, and gravitation of blood, were the two different conclusions. A portion of the lung thus filled with blood, was taken out, and put in water—it seemed as sound and light, after repeated trials, as any other less coloured part.

The abdomen was then inspected. The liver appeared sound in its aspect and size, but along its outer edge it seemed of a darker hue than the other portions. Here congestion was again seen by two of the gentlemen, but denied to exist by the three others.

Then came the stomach, which, internally and externally, was free from disease. The small intestines were found, to the extent of two feet, inflamed on their mucous surface—the blood was exhaled from the mucous tissue, and coated it completely. This state of the intestinal tube was called venous congestion by two of the gentlemen, the other three argued that phlogosis evidently existed; that the mere loss of blood from the bowels was not sufficient to destroy the patient; but that the injury done by a highly acute state of inflammation, in a part so important, had terminated life. Every other part of the intestines was sound. For reasons the reader can easily appreciate, I avoid any disclosure of names. We cultivate a profession in which all the generous feelings of our nature should have exercise, and to avoid all unnecessary occasions of inflicting a wound on the most susceptible sensibility is our bounden duty.

The fourth pathological state with which venous congestion may be confounded, is that condition of the system, denominated *collapse.*

After the excitement of bilious fever has continued several days, or weeks, according as the powers of resistance may be in the patient, the action of the heart becomes enfeebled, the pulse, instead of being tense and quick, or full and slow, gradually sinks down into a soft, compressible, and more rapid stroke; the skin loses its fervent temperature, and assumes a more natural heat, or becomes cold and clammy; the tongue is dry and brown; the alvine discharges are watery and frequent, or they are dark coloured and small; the eyes become blood-shot, or weak and watery; the patient lies on his back, with his knees drawn up; there is some subsultus of the fore arms, felt when you grasp the wrist, though slight at first; the eyes are rolled up, as if the patient were asleep, but the lids do not cover them, so that you see their white part; and when asked how he is, he answers, "pretty well," though his mind, unless roused by your interrogation, seems incoherent, or comatose.

Now, a physician, whose head was surcharged with the doctrine of venous congestion, would fasten on these symptoms as indicative of that state of the vessels. But if he attempted to treat the patient on this principle, the plan of cure would prove a quick acceleration of his death.

I speak from a personal trial of the therapeutic procedure, which is built on the false foundation of venous congestion in the typhoid state of fever. This collapse of the system may exist, without any organic læsion whatever, and most generally is unaccompanied by structural alterations. For if inflammation existed in any important organ, the case would terminate more speedily, and the recoveries would not be so common as they are under a proper treatment. Besides, were there inflammation, the symptoms would indicate it in a less equivocal manner.

The nervous system is to be adverted to, as affording a clue to guide our steps through the intricate perplexities of such cases. Mere loss of balance in the distribution of the blood does not satisfactorily obviate the difficulty. Effects should not be substituted for causes, nor should one symptom be allowed to fill the entire field of our intellectual vision. The symptoms point to some *functional* derangement of the nervous system, connected with an arrest of certain important secretions, such as those of the skin, liver, and kidneys. And the inspection of the dead body confirms this view. The following case is in point; it is selected from several others of a similar character.

A man, of about twenty years of age, addicted to drinking whiskey, of a thick well set frame, and of full habit, was brought into the Louisville Hospital, from the Louisville and Portland Canal, affected with measles. He was bled and purged liberally, and had been treated properly in every respect by Dr. Hall. In two weeks from his admission he died; before which, he had low delirium, and other symptoms of a collapsed state of the system. *Sectio cadaveris*, two hours after death. Not a single trace of disease could be discovered either in the brain, or viscera of the thorax and abdomen. A more natural exhibition of the organs I have never seen.

It is, perhaps, worse than useless, to speculate on such cases, the reader must exercise his own powers of ratiocination, or it is in vain that the above facts are spread before him.

A few practical reflections and inferences will close this paper.

The central point at which all the rays of our information on medical subjects should meet, is the acquisition of a sound, discriminating, practical judgment. A judgment which will enable us to stand prepared and ready for decisive measures in the critical hour of a fellow being's sufferings and peril. To assist in the formation of this clinical discernment, I would ask my reader, whether he does not see that there is in pathology, no such a state of the system as venous congestion.

In the next place, is there not room for careful perquisition into every case, however at first sight similar, afforded in the views given above. 1st, on simple irritation, inducing sympathetic affections. 2d, Constitutional shock, putting on a semblance of venous congestion, as described by Dr. A. 3d, Suffocated excitement, which is the real pathology of the cases detailed by Dr. A. and 4th, Collapse of the system, after the vires vitales have been prostrated by continued febrile orgasm.

I have tried to avoid excessive refinement in the views presented. Subtle and wire drawn distinctions may amuse in the closet, but they avail nothing at the *bed side.* There the mind must, with comprehensive discernment, see the case in its predominant and ruling features. To ascertain the real nature of the case under inquisition, we must attend to its history; the mode of seizure; the habits of the patient's life; the constitutional tendencies of the individual, whether he, or she, is of an irritable nervous temperament; or sanguineous and liable to inflammatory affections; and whether there has been any medical treatment previously pursued.

The treatment of sympathetic derangement should be guided and directed by a knowledge of the cause giving origin to such aberration from health. It will be found that in a majority of such cases the intestinal canal is the seat of the morbid irritation. Purgatives, especially mercurial, in such instances are the best means of administering relief.

The fond reveries of a system monger may exclude effective cathartic remedies from our therapeutics, but a well disciplined practical judgment holds on its way, unmoved from its decisions by such false lights.

The uterus in women, sometimes, gives rise to sympathetic affections, of a hysterical kind, these are to be treated by purgation, which creating an impression on a different quarter, diverts irritation from the womb. Hysteria, most frequently, originates from the stomach and bowels, and therefore catharsis is more demanded.

Dr. Marshall Hall's work on Mimoses, is a further confirmation of Hamilton's practice, aud powerfully illustrates the sympathies that bind the stomach and bowels to every part of the human body.

In cases of shock, or constitutional irritation, from concussion, or a severe accident, our duty is to wait for reaction, before we officiously begin to deplete. Surgeons well understand this important step in treating such cases, yet I have known even grey headed physicians open a man's vein, whilst lying on the spot where he received the injury, cold and nearly pulseless.

In all such cases the plan of treatment is to put the patient to bed; apply warm bricks to his extremities and under his arms; give warm teas if he can swallow; and wait the issue. In cases of burns, laudanum is generally prescribed, though Mr. Travers condemns it, and prefers giving a spoonful of brandy every hour or two, in two spoonsful of gruel. When reaction is restored, he withdraws the stimulus.

In suffocated excitement, where the actions are imprisoned under the strong urgency and excessive force of disease, we must watch every alteration with eagle-eyed regard. Blood-letting opportunely executed, is a valuable mean of developing action. But purgative medicines are a safer resource. Drs. Rush, Armstrong, and Johnson, have written on this practical point, what I will not attempt to re-write—to them the reader should go for a triumphant vindication of the depletory practice, and for a matter of fact refutation of the erroneous practice pursued by some physicians in analagous cases.

Dr. Potter, in a note to Armstrong on Typhus, admits that "the symptoms so formidable at the close of fevers are often nervous only, and the seeming congestions of as much importance as they are in hysteria."—p. 300. Accordant with this view, we should sustain the faltering efforts of life, by well adapted excitants, such as camphor, opium, vol. alkali, &c. Mercurial and antimonial preparations are to be used in very minute quantities, merely to promote the secretions. All purgative medicines must be avoided.

Wine, wine whey, panada, thin soups &c. are required, the feeble powers however, must not be goaded on to excessive action, but the sinking, quivering flame must be kept alive by a gradual supply of needful fuel.

Blisters, or sinapisms, to the extremities are useful, but we must not suffer them to remain on too long, otherwise much injury may be produced by the exhausting irritation which they excite.

I must stop—though the subject branches out the more my mind contemplates it, in its high bearings and diversified applications. To the reader I appeal for a candid scrutiny of the foregoing observations, and I confidently trust, that they may minister some aid to the inquiring mind in its search after truth, and contribute, in some degree, to the improvement of a profession, in which it is my daily occupation to be engaged, both as a necessary avocation, and as a source of elevated mental satisfaction.

*Louisville, November* 8, 1828.

